# An Ultrasound-Based Preoperative Evaluation of the Endometriosis Fertility Index: A Further Step towards Personalized Treatment

**DOI:** 10.3390/jcm13051488

**Published:** 2024-03-05

**Authors:** Matteo Marchetti, Marco Noventa, Eleonora Panizzolo, Valentina Pianon, Matteo Tamagnini, Sofia Bigardi, Carlo Saccardi, Roberto Tozzi, Giulia Spagnol

**Affiliations:** Unit of Gynecology and Obstetrics, Department of Women and Children’s Health, University of Padua, 35100 Padua, Italy; matteomarchetti91@gmail.com (M.M.); eleonorapanizzolo2@gmail.com (E.P.); vale.pianon@gmail.com (V.P.); matteo.tamagnini.1991@gmail.com (M.T.); sofiabigardi8@gmail.com (S.B.); carlo.saccardi@unipd.it (C.S.); roberto.tozzi@unipd.it (R.T.); giuliaspagnol.ts@gmail.com (G.S.)

**Keywords:** endometriosis, EFI, classification, infertility, ultrasound, laparoscopy, preoperative assessment

## Abstract

**Background**: The Endometriosis Fertility Index (EFI), is a crucial validated surgical tool used for predicting fertility outcomes in women with endometriosis. This study aims to assess the concordance between a preoperative clinical and instrumental EFI evaluation (uEFI) and the EFI score obtained during an exploratory laparoscopy prior to surgery (sEFI). **Methods**: This study presents preliminary data from a broader observational cohort study. The Least Function score for the uEFI was calculated using a modified version of the original surgical EFI by incorporating a clinical examination, advanced ultrasound, and hysterosalpingo-foam sonography (HyFoSy). **Results**: The preoperative estimation of the EFI (uEFI) demonstrated a high concordance (k = 0.695, ***ρ***_s_ = 0.811) with the sEFI. Remarkably, the surgical interventions led to a significant improvement in the EFI values, with 80% of the intermediate EFI transitioning to a high level, thereby highlighting the positive impact of surgery on fertility outcomes. **Conclusion**: This study highlights the accuracy of preoperative EFI estimation (uEFI) and its strong agreement with intraoperative assessment. It underscores the potential of a preoperative management tool to guide the allocation of infertile women with endometriosis to operative laparoscopy, direct assisted reproductive technology (ART), or spontaneous attempts at pregnancy.

## 1. Introduction

Endometriosis is a complex and debilitating gynecological disorder that frequently impacts pelvic structures, affecting 6–10% of women of childbearing age, with a total of about 190 million people being affected worldwide [1]. Endometriosis is associated, in a high percentage of cases, with infertility; estimates suggest that roughly 30–50% of women diagnosed with endometriosis face challenges in achieving pregnancy [2]. The intricate link between endometriosis and infertility involves various mechanisms, but a distortion of the pelvic anatomy, termed the “pelvic factor”, emerges as a predominant element, especially in severe forms of endometriosis. Pelvic or peritubal adhesions can compromise the release, collection, and transport of oocytes, contributing to infertility [3].

Although surgery seems to be an obvious therapeutic choice, the scientific evidence supporting its effectiveness in treating endometriosis-related infertility is limited. Despite some studies indicating increased reproductive chances with surgical treatment, comparative studies directly assessing spontaneous conception probabilities before and after a surgical excision of deep endometriosis lesions are lacking [4,5,6].

In 2010, Adamson and Pasta introduced the Endometriosis Fertility Index (EFI) as a valuable clinical tool for predicting fertility potential in women with endometriosis [7]. The EFI score aims to forecast the postoperative pregnancy rate (PR) in patients with surgically confirmed endometriosis, attempting conception without assisted reproductive technology (ART) [7,8]. The 10-point EFI incorporates three *historical factors* (patient’s age, duration of infertility, and pregnancy history), contributing a maximum of 5 points, and three *surgical factors* estimating residual adnexal function and the extent of endometriosis, collectively contributing the remaining 5 points. The surgical factor score is derived from three distinct values: the *Least Function Score (LF Score),* characterizing adnexal functionality post-surgery; the American Fertility Society *(AFS) Endometriosis Score* based on lesion size following the revised American Society for Reproductive Medicine (rASRM) classification; and the *AFS Total Score*, extending the *AFS Endometriosis Score* to include additional factors like tubal and ovarian adhesions and the obliteration of the Douglas pouch [7,9]. The EFI score ranges from 0 to 10, with an improved fertility prognosis being associated with a higher final score. Notably, a recent meta-analysis confirmed that the EFI score could successfully predict the non-ART pregnancy rate after laparoscopy: women with a high EFI score had a higher chance of a non-ART pregnancy compared with women with a low EFI score; in addition, it showed that the EFI demonstrated superior predictive power compared to rASRM for the successful prediction of PR, irrespective of ART, in women with endometriosis [10,11].

Despite its significant role in predicting post-intervention fertility rates for endometriosis patients, the EFI remains a tool for post-surgical intervention; it is not universally applicable to all cases of infertile endometriosis patients and it poses a risk of surgical overtreatment. This prompts the question of whether the EFI can be determined preoperatively based on clinical presurgical data to guide patients on the optimal management of their infertility. In this context, a 2020 article by Tomassetti et al. demonstrated a high level of agreement between pre-operative ultrasound assessments and intraoperative laparoscopic findings in centers specialized in the diagnosis and treatment of endometriosis. This suggests that, for the personalized treatment of women with endometriosis-related infertility, the EFI could serve as a supportive tool in decision making between surgery, ART, or other reproductive management approaches [12].

Our study presents promising preliminary data derived from a broader prospective investigation aimed at evaluating the efficacy of laparoscopic surgery for endometriosis-related infertility in improving fertility outcomes. The primary objective of the current study is to assess the feasibility of accurately estimating the Endometriosis Fertility Index through non-invasive clinical and instrumental investigations prior to surgery. This approach aims to facilitate non-surgical management strategies for patients and optimize the directions of their reproductive paths.

## 2. Materials and Methods

### 2.1. Study Design

The current investigation constitutes a preliminary analysis derived from an ongoing prospective observational cohort study conducted at a singular institution, the Obstetric-gynecological Department of the University Hospital of Padua, involving women diagnosed with endometriosis. This study evolved based on data collected from patients who underwent clinical and instrumental examinations in our clinics and subsequent surgical procedures between January 2022 and December 2023. This study was approved by the ethics committee of the University Hospitals of Padua with the following protocol number: 457N/AO/2022. The primary objective of this investigation is to assess the feasibility of accurately estimating the EFI in women experiencing endometriosis-related infertility even before undergoing surgery. This estimation relies on non-invasive clinical and instrumental assessments, such as ultrasound and hysterosalpingo-foam sonography (HyFoSy), with the intention of deriving benefits from non-surgical management strategies for these patients.

### 2.2. Study Population

We selected women of childbearing age, aged between 18 and 42, who were referred to the “Chronic Pelvic Pain” outpatient clinic at our institution, with clinical and ultrasound diagnoses of endometriosis and the presence of infertility, defined as the absence of conception after 12 months of regular complete unprotected sexual intercourse, in the absence of other known causes of infertility.

The exclusion criteria were age <18 or >42, no desire for pregnancy, seeking pregnancy directly through ART, surgery at another facility, attempting pregnancy for less than 12 months, concurrent gynecological conditions contributing to infertility (polycystic ovary syndrome (PCOS), submucosal fibroids, secondary amenorrhea due to other causes, pelvic inflammatory disease, or uterine malformations), male infertility of the partner, and refusal to provide informed consent.

### 2.3. Outcomes of the Study

The primary outcome of our study was assessing the concordance rate between the preoperative clinical and instrumental evaluations of the EFI (ultrasound EFI or uEFI) and the EFI score obtained during exploratory laparoscopy conducted at the time of surgery (surgical EFI or sEFI).

Secondary outcomes included the rate of agreement between uEFI and the EFI score calculated at the end of surgical laparoscopic procedures, referred to as post-surgical EFI or psEFI; identification of EFI clinical classes for which surgery can be avoided, as they are unnecessary or ineffective in order to propose a management algorithm; fertility outcomes (rate of conception and pregnancy brought to term), both spontaneous or through ART, along the 12-month follow-up of the patients.

### 2.4. Data Collection and Procedures

All essential data for the present study were collected and stored in a research database, in an anonymous form, linked solely through a hospital identification number to ensure confidentiality. All patients were interviewed for collection of medical records and clinical data to fill in the historical factors form, accounting for the first 5 points of the EFI. Subsequently, all of them underwent gynecological abdominal and bimanual examination, transvaginal ultrasound (TVUS), and HyFoSy. Finally, the patients underwent laparoscopic surgical procedures performed by three experienced surgeons (M.N., C.S., and R.T.) in accordance with the guidelines of the European Society of Human Reproduction and Embryology (ESHRE) [6]. The surgery, tailored to each individual case, involved extensive adhesiolysis to free all pelvic structures, the removal of any endometriomas while preserving as much healthy ovarian tissue as possible, and the release of the fallopian tubes from any adhesions or nodules, with salpingotomy or salpingectomy performed in cases of documented tubal occlusion. As it was reproductive surgery, any deep endometriosis nodules not directly involving pelvic organs were excised only in cases of moderate to severe symptoms refractory to medical treatment. During surgery, a check of tubal patency was conducted in all patients by visualizing the passage of methylene blue through the tubes, which had been previously injected into the cervical canal (chromopertubation test). All patients signed an informed consent form for the use of their clinical information in anonymous form for scientific research purposes.

TVUSs were performed by two high-level operators specialized in deep infiltrating endometriosis (DIE) using Samsung Hera W9 (Samsung Medison Co., Ltd., Seoul, South Korea) or GE Voluson E10 ultrasound (GE Healthcare, Zipf, Austria) equipped with a 7.5–9 MHz transvaginal probe, and reports were generated following the IDEA protocol [13]. HyFoSy was conducted using a gel foam (ExEm Foam—Unimed Srl., Pordenone, Italy) as a contrast medium, introduced by placing a 5 Fr HyFoSy balloon catheter in the uterine cavity and filling it with 1–2 mL of water to prevent fluid leakage from the cervix. A 2D real-time ultrasound, followed by a 3D reconstruction, where possible, was performed to assess tubal patency.

For each patient, EFI estimations were performed by carrying out rating at three different time points:

- Ultrasound EFI (uEFI) was computed by integrating medical historical data obtained during the outpatient examination with a modified model derived from the one proposed by Tomassetti et al. [12], as illustrated in Table 1. Fimbria evaluation was deemed not possible through pre-surgical instrumental exams. Consequently, the Least Function (LF) score was calculated as the sum of the lowest score between ovarian and tubal function from each side. The AFS Endometriosis Score and AFS Total Score were instead calculated according to the original model developed by Adamson and Pasta [6] based on the revised classification of the ASRM.
- Surgical EFI (sEFI) was calculated at the time of exploratory laparoscopy, i.e., upon introduction of the scope prior to surgical intervention.
- Post-surgical EFI (psEFI) was assessed after surgery and describes the state of the pelvis after adhesiolysis and removal of all macroscopically visible endometriosis lesions.

As they represent intraoperative scores, the sEFI and psEFI were obtained according to the original model described by Adamson and Pasta [6] by integrating medical historical factors previously collected for uEFI (historical factors) with data obtained during exploratory laparoscopy. Tubal patency, required for the calculation of the LF score, was assessed using the chromopertubation test with methylene blue.

As previously proposed by Tomassetti et al. [12], the EFI values obtained were categorized into 3 subclasses based on the fertility outcome rates, i.e., the probability of achieving spontaneous pregnancy, historically described in [7,10]. EFI scores ≤ 4 were considered low, and scores of 5–6 were considered as intermediate, while those ≥7 were considered to have a high probability of spontaneous pregnancy.

### 2.5. Statistical Analysis

Data were analyzed using SPSS 22.0 software (SPSS Inc., Chicago, IL, USA). Descriptive variables (continuous variables) were summarized as median and interquartile range (IQR) or frequency and percentage (categorical variables).

We analyzed ordinal variables using Kruskal–Wallis test. Categorical variables were expressed as percentage and analyzed through the chi-squared (χ^2^) test or Fisher’s test when appropriate. The level of correlation and agreement between uEFI, sEFI, and psEFI was evaluated with Cohen’s weighted Kappa and Spearman rank correlation. Statistically significant differences were defined as *p*-value < 0.05.

## 3. Results

### 3.1. Patient Selection and General Characteristics

During the study period, 197 patients were referred to our “Chronic Pelvic Pain” outpatient clinic, and 165 of them received a clinical–instrumental diagnosis of endometriosis. Only infertile patients expressing a desire for pregnancy and actively seeking spontaneous conception were deemed eligible for the present study.

**Figure 1:**
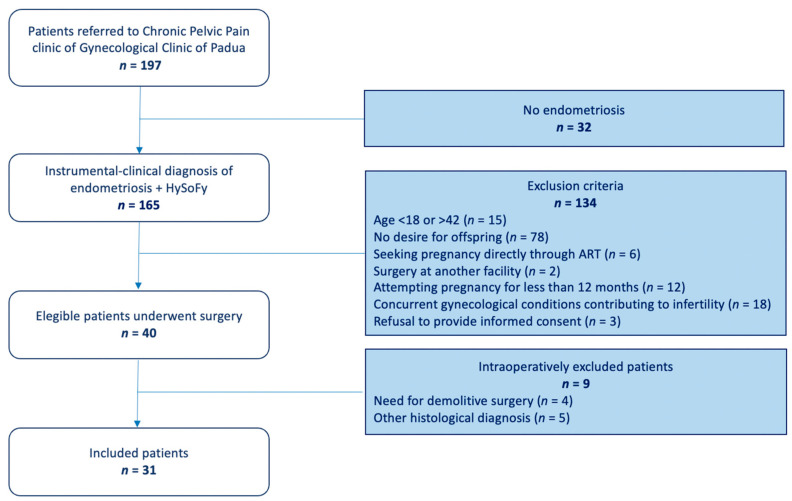
Flowchart of patient selection for this study.

Thirty-one patients were included for the analysis. The selection flow diagram of this study is shown in Figure 1.

General demographic features were used to fill in the historical factors of the EFI score and included a median age of 34 years old [IQR: 29.00–40.00]. A total of 7 patients (22.6%) achieved prior spontaneous pregnancies, while 24 (77.4%) were nulliparous.

Lastly, among the 31 women considered, 25 (80.6%) had unsuccessfully attempted spontaneous pregnancy for more than 3 years. In Table 2, the baseline symptoms related to endometriosis and the disease localizations identified during clinical and intraoperative examinations are presented.

All patients in our cohort were symptomatic, exhibiting at least one symptom related to endometriosis. The tubal patency information obtained preoperatively through HyFoSy documented 5 patients with bilateral fallopian tube closure and 4 with a unilateral occlusion of a tube, while bilateral tubal patency was present in the remaining 22 patients.

### 3.2. Ultrasound, Surgical, and Post-Surgical EFI Scores

The median value of the EFI score assessed during the preoperative II-level pelvic ultrasound (uEFI) was found to be 6 [IQR: 5–7], and the intraoperative pre-intervention (sEFI) value was also found to be 6 [IQR: 5–7], while at the end of the procedure (psEFI), it was 8 [IQR: 6–9].

**Figure 2:**
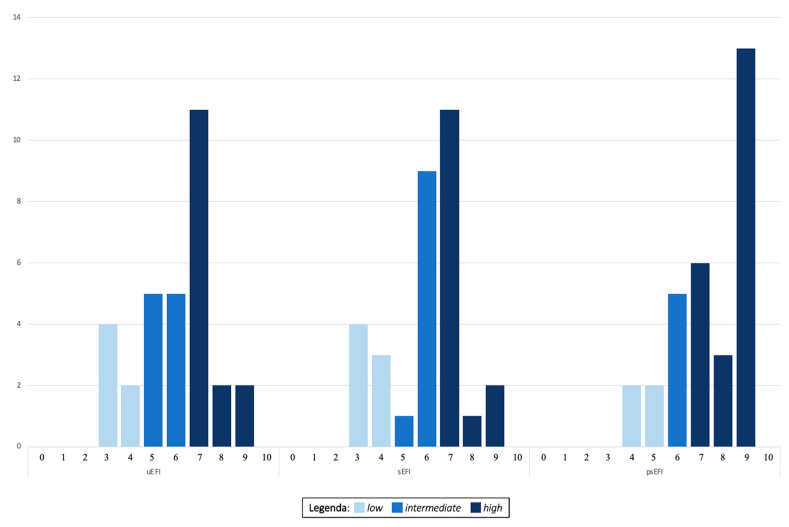
EFI values obtained at three different time points in our population: presurgical ultrasound-based EFI (uEFI), intraoperative EFI at time of exploratory laparoscopy (sEFI), and post-procedure EFI score (psEFI). X-axis represents EFI values; y-axis indicates number of patients with that value.

Figure 2 shows the distribution of the different EFI scores in our population: in all three models, the majority of the patients had EFI scores found in the high range of value (EFI 7–10), particularly at the post-surgical evaluation (uEFI 15/31 (48.3%), sEFI 14/31 (45.1%), and psEFI 22/31 (70.9%)). The number of patients exhibiting intermediate values (EFI 5–6) was slightly lower than the previous assessments in pre-surgical EFI evaluations (uEFI 10/31 (33.3%) and sEFI 10/31 (33.3%)) and significantly lower in psEFI (22.5%). A small proportion of patients had EFI scores in the low range of value (EFI 0–4) at all three analyses (uEFI 6/31 (19.3%), sEFI 7/31 (22.5%), and psEFI 2/31 (6.4%)).

An analysis of variance conducted through the Kruskal–Wallis test revealed no statistically significant differences between the uEFI and sEFI scores (*p* = 0.961). However, a statistically significant difference was observed between uEFI and psEFI, as well as between sEFI and psEFI (*p* = 0.002 in both cases).

In Table 3, the results of the correlation analyses conducted among the EFI scores at the three previously defined time points are presented, including the analyses performed for the subcategories (low, intermediate, and high). A concordance analysis between clinical classes uEFI and sEFI showed a good Cohen’s Kappa (range 0.6–0.8) with k= 0.634, and the value increased to k = 0.695 when comparing the data based on single categories (*low, intermediate,* and *high*), with a Spearman rank correlation of ***ρ***_s_ = 0.811.

It is noteworthy that among the six patients with a low u-EFI value, two (33.3%) maintained the same value, and four (66.7%) transitioned to an intermediate value, but none increased their EFI to a high level. Conversely, among the 10 patients with an intermediate u-EFI, 2 (20.0%) maintained the same value, none worsened, while the remaining 8 (80%) transitioned to a high score.

### 3.3. Fertility Outcomes

The study patients underwent a median follow-up of 12 months. Eighteen out of thirty-one patients chose to delay the pursuit of pregnancy for personal reasons and opted to resume hormonal medical therapy (combined estrogen-progestin or progestin-only). Thirteen patients, on the other hand, initiated attempts to achieve spontaneous pregnancy: five (38.4%) successfully conceived, while eight (61.6%) were unsuccessful (one of whom chose to discontinue the period of spontaneous pregnancy attempts and directly initiate ART techniques). Among the latter, four patients (30.7%) achieved pregnancy through ART, and another four are beginning the assisted reproduction process.

## 4. Discussion

The EFI score has now become a validated tool for predicting fertility outcomes, particularly the likelihood of successful spontaneous pregnancy in patients with endometriosis [10]. The inclusion of historical factors and the emphasis on the functional assessment of the tubes and ovaries, rather than a subjective definition of disease spread, make EFI more functional for this purpose compared to the rASRM classification [9]. However, the EFI score is primarily designed as a post-operative score and may not be well suited for counseling a patient desiring pregnancy with a new diagnosis of endometriosis.

Tomassetti et al. [12] were the first to demonstrate a concordance of 0.915 between preoperative clinical and ultrasound assessments of the EFI (referred to as EFI type A) and intraoperative evaluation (type B). However, they showed a lack of improvement in the final post-surgical EFI score. The primary objectives of the present study were precisely these two, namely the concordance between uEFI and sEFI and the assessment of potential improvement in the score in patients undergoing surgical intervention (psEFI). In our study, we present preliminary data from a validation study of the preoperative EFI based on clinical information, a comprehensive second-level pelvic ultrasound (TVUS) for the functional assessment of endometriosis, and HyFoSy (uEFI).

**Figure 3:**
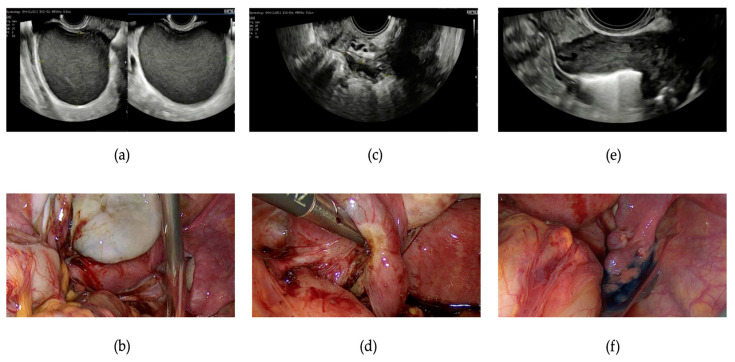
Presurgical and intraoperative images of a patient who underwent laparoscopy for ovarian and deep infiltrating endometriosis, illustrating the agreement between the ultrasound and surgical findings. (**a**) An ultrasound image depicting a typical endometrioma of the left ovary with a characteristic ground glass appearance; (**b**) a laparoscopic view of the left ovarian endometrioma; (**c**) an endometriotic nodule of the left parametrium, causing stretching of the ipsilateral tube and adhesions between the ovary, tube, and bowel; (**d**) an intraoperative view of the left tube, which is dilated, convoluted, and adhered to the ovary and peritoneal nodule. Subsequent chromopertubation will confirm the lack of patency of the left tube. (**e**) Hysterosalpingo-foam sonography (HyFoSy) of the right tube demonstrating the patent status on a presurgical examination. (**f**) Chromopertubation with methylene blue confirming the patency of the right tube during surgery.

The data, albeit preliminary, on the concordance between uEFI and sEFI clearly indicate a high agreement (k = 0.7) between the preoperative assessment and laparoscopic evaluation, suggesting, in line with the latest ESHRE guidelines, that laparoscopy should be avoided solely for diagnostic purposes [6]. It is essential to emphasize the crucial role of specialized sonographers in the diagnosis and evaluation of endometriotic pathology, especially in cases with deep infiltrating endometriosis [15] (as seen in Figure 3), supporting the centralization of patients with endometriosis seeking pregnancy in specialized centers, as practiced in our center (following the terms and definitions of the IDEA-group [13]). This represents an initial limitation to the widespread adoption of preoperative EFI across all facilities, as also highlighted in the previous article on this topic by Tomassetti et al. [12]. Similar to the aforementioned article, we report a nearly identical predictive capacity for fertility outcomes between preoperative and surgical analyses.

However, a second important result from our study, diverging from previous reports by colleagues, is the significant improvement in the EFI score that was achieved through a surgical intervention, particularly in patients diagnosed with *intermediate* (of which 80% transitioned to a *high* score) or *high* uEFI scores. These data are further noteworthy considering the inherent nature of the EFI score, which includes an unmodifiable historical component and only half of the final result that is amenable to improvement through intervention. According to the now validated curves of the score proposed by Adamson et al. [7], the enhancement in fertility outcomes at 12 months is particularly evident for EFIs > 6, specifically for scores ≥ 9, with a pregnancy rate of 60% at 12 months. Unfortunately, the limited sample size did not allow for a further subdivision of the *high* class EFI into two subgroups, namely 7–8 and 9–10. However, as easily observed from Figure 2, a significant improvement in the EFI score occurred after the surgical intervention. In fact, while only 2 patients had a value ≥ 9 in both uEFI and sEFI, a total of 13 patients showed an EFI of 9 at the end of the surgery. Our objective, as the study progresses, is to conduct a more extensive subgroup analysis to confirm this finding.

**Figure 4:**
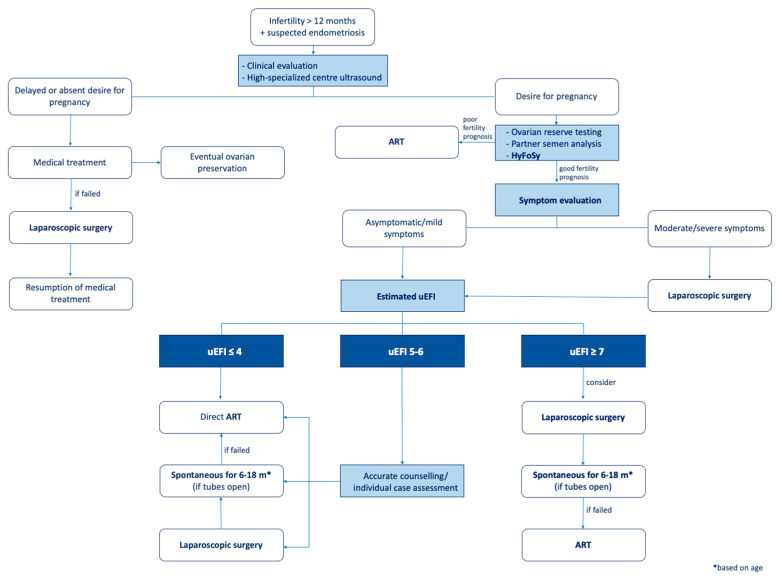
Possible management flowchart of infertile endometriosis patients dependent on the uEFI value. ART, assisted reproductive technology; HyFoSy, hysterosalpingo-foam sonography; uEFI, ultrasound EFI; m, months.

As a consequence of the promising initial results from this preliminary study, we advocate for the application of a preoperative EFI in counseling patients with endometriosis seeking pregnancy, always comprising TVUS (performed by a highly skilled and experienced operator) in combination with HyFoSy to assess tubal patency (i.e., Figure 3). In Figure 4, an algorithm proposed for the management of these patients is illustrated. Patients with an EFI score ≤ 4 will be directed straight to an ART pathway; the recommendation for surgical intervention in these cases is reserved only for endometriosis symptoms that are not resolvable with medical therapy alone. For cases with EFI ≥ 7, surgery at specialized centers may represent a favorable option given the potential for increased EFI scores, thereby improving fertility outcomes. Following surgery, a period of 6–18 months for attempting spontaneous pregnancy, adjusted according to the patient’s age, is recommended. However, for cases with *intermediate* uEFI (5–6), the approach becomes more nuanced and necessitates individual assessment; surgery followed by attempting spontaneous pregnancy for 6–18 months may still represent a viable option. Indeed, as described previously, we observed an 80% improvement rate in the EFI values following surgery, transitioning from *intermediate* to *high*. Otherwise, a direct ART pathway could represent a valid solution in any case. However, the decision-making process underlying the newly proposed algorithm needs to be further explored to achieve true customization. Factors such as patient age stratification and an assessment of the ovarian reserve are believed to be necessary inclusions and should therefore be considered in the near future. Indeed, in the case, for example, of a patient with endometriosis and poor ovarian reserve, clinically performing HyFoSy to assess the uEFI would be clinically futile and even harmful from a cost-effectiveness standpoint, as the path to second-level ART would be necessary regardless and should be undertaken as soon as possible.

Our data, although certainly encouraging both in terms of the preoperative prediction of the EFI score and the improvement achieved by surgery in the hands of experienced surgeons, remain preliminary and require further studies. The continuation of this study will be crucial for the standardization and validation of this preoperative tool to enhance counseling and management for infertile patients with endometriosis.

Regarding the fertility rate in our sample, the data acquired so far are still too premature, and the follow-up is too narrow to draw conclusions, which represents a limitation of our study, even though the correlation between the psEFI and fertility outcomes is now validated and not the primary outcome of the present study. Additional and more decisive information regarding this topic is expected to be derived from the continuation of our prospective study.

## 5. Conclusions

In conclusion, our study confirms that the EFI score can be accurately predicted (uEFI) through a combination of clinical examination, advanced ultrasound, and HyFoSy in a preoperative setting. This underscores the potential of a preoperative management tool to guide the allocation of infertile women with endometriosis to operative laparoscopy, direct ART, or spontaneous attempts at pregnancy. Nevertheless, this study highlights the crucial role of surgery in enhancing the preoperative EFI score and, consequently, the fertility outcome in these patients.

## Figures and Tables

**Table 1:** Pre-operative adapted model for calculating LF score in the uEFI.

Structure	Functionality	Description
Tube	4 Normal	Tube promptly patent, linear tubal course
	2 Moderate	Tube slowly patent or patent only after second injection
	0 Nonfunctional	Closed tube
Ovary	4 Normal	Normal ovarian size, follicles present
	3 Mild	Endometrioma < 3 cm, positive *crescent sign* *
	2 Moderate/severe	Endometrioma ≥ 3 cm, positive *crescent sign* *
	0 Nonfunctional	Any endometrioma, no *crescent sign* *

* Ovarian *crescent sign*, described by the International Ovarian Tumor Analysis (IOTA) group as “a rim of normal ovarian tissue adjacent to an ipsilateral adnexal lesion” [14].

**Table 2:** Endometriosis symptoms; clinical/instrumental pre-operative assessments. Total population = 31. CPP, chronic pelvic pain; LBP, low back pain; TVUS, transvaginal ultrasound; USLs, utero-sacral ligaments; RVS, recto-vaginal septum; HyFoSy, hysterosalpingo-foam sonography.

	*n*/Total (%)
**Symptoms**	
Dysmenorrhea	23/31 (74.2)
Dyspareunia	15/31 (48.4)
CPP	12/31 (38.7)
LBP	9/31 (29.0)
Dysuria	3/31 (9.7)
Dyschezia	6/31 (19.4)
**Gynecological Exam + TVUS**	
Unilateral ovarian cyst	20/31 (64.5)
Bilateral ovarian cysts	6/31 (19.3)
”Kissing ovaries”	11/31 (35.5)
Deep Infiltrating Endometriosis *	15/31 (48.4)
USLs	10/31 (32.3)
RVS	4/31 (12.9)
Parametria	5/31 (16.1)
Sigmoid colon/rectum	1/31 (3.2)
Bladder	-
Skin	1/31 (3.2)
Inguinal canal	1/31 (3.2)
**HyFoSy**	
Bilateral patency	22/31 (70.9)
Unilateral patency	4/31 (12.9)
Closed tubes	5/31 (16.1)

* Possibly more than one localization.

**Table 3:** General and categorized agreement between EFI and two surgical EFIs (sEFI and psEFI).

	uEFI vs. sEFI	uEFI vs. psEFI
**General**		
Correlation (95% CI) *	0.87	0.79
Weight Kappa (95% CI)	0.64	-
**By category**		
Correlation (95% CI) *	0.81	0.67
Weight Kappa (95% CI)	0.70	0.22
**Concordance by category**		
*Low*	1.00	0.33
*Intermediate*	0.70	0.20
*High*	0.80	0.93

* Correlation is expressed through Spearman ***ρ***_s._

## Data Availability

Data are available upon request.
